# Laser-Assisted Treatment for Maxillary Peripheral Odontogenic Fibroma: A Case Report

**DOI:** 10.7759/cureus.59453

**Published:** 2024-05-01

**Authors:** Ruchita T Patil, Prasad V Dhadse, Shrishti S Salian, Sanehi D Punse

**Affiliations:** 1 Department of Periodontics and Implantology, Sharad Pawar Dental College, Datta Meghe Institute of Higher Education and Research, Wardha, IND

**Keywords:** recurrence risk, excision surgery, histological features, anterior maxilla, fibroma

## Abstract

Most odontogenic tumors are intraosseous growths. A peripheral odontogenic fibroma presents as a slow-growing and firm swelling on the gingiva. It develops more commonly on the mandibular than the maxillary region. It can be found on either the palatal or lingual and on the labial or buccal surface of the jaw. It usually does not ulcerate. The most common type is a peripheral odontogenic fibroma, which is a benign odontogenic neoplasm of the periodontal soft tissues. In this case report, a 53-year-old male patient with peripheral odontogenic fibroma was treated using a laser.

## Introduction

Central odontogenic fibroma (COF) is an exceptionally rare benign tumor. It is characterized by a fibrous mature stroma containing strands or islands of inactive-looking odontogenic epithelium. Less than 0.1% of oral lesions and 1.5% of all odontogenic malignancies are caused by COF. It affects people of all ages, from four to 80, with a minor tendency toward females over males. Recent research disproves the initial theory of mandibular predominance and shows that COF occurs in equal frequency in both the mandible and maxilla. It usually appears in the maxilla's anterior and the mandible's posterior region [1].

In 2005, the World Health Organization (WHO) distinguished between two histological forms of COF: the complex type, which has a large number of epithelial cells, and the simplex type, which has a few epithelial islands. Odontogenic epithelial nests and dispersed mature tissue resembling cementum, osteoid tissue, or dysplastic dentin are seen in the epithelium-poor variety of ossifying fibroma. On the other hand, the epithelium-rich version has more epithelial nests and is more cellular [2]. COF appears radiographically as a distinct, radiolucent lesion that may be unilocular or multilocular. In addition, it usually grows slowly within the bone without affecting the cortical outline and preserving the anatomical structures. Root divergence and recurrent resorption of the adjacent teeth are frequently caused by lesions that arise in between the teeth [3]. This case report demonstrates the excision of peripheral odontogenic fibroma in the maxillary esthetic region using a laser diode.

## Case presentation

A 53-year-old male patient reported to the Department of Periodontics and Implantology, with a chief complaint of swelling and pain in the upper front region of the jaw for three to four months. The swelling was progressively increasing in size. No associated past medical history or systemic condition was present. No gross asymmetry was observed during the extraoral examination, and there was bilaterally smooth and synchronized temporomandibular joint movement. The submental and submandibular lymph nodes on both sides were inspected, and no significant history was recorded.

**Figure 1:**
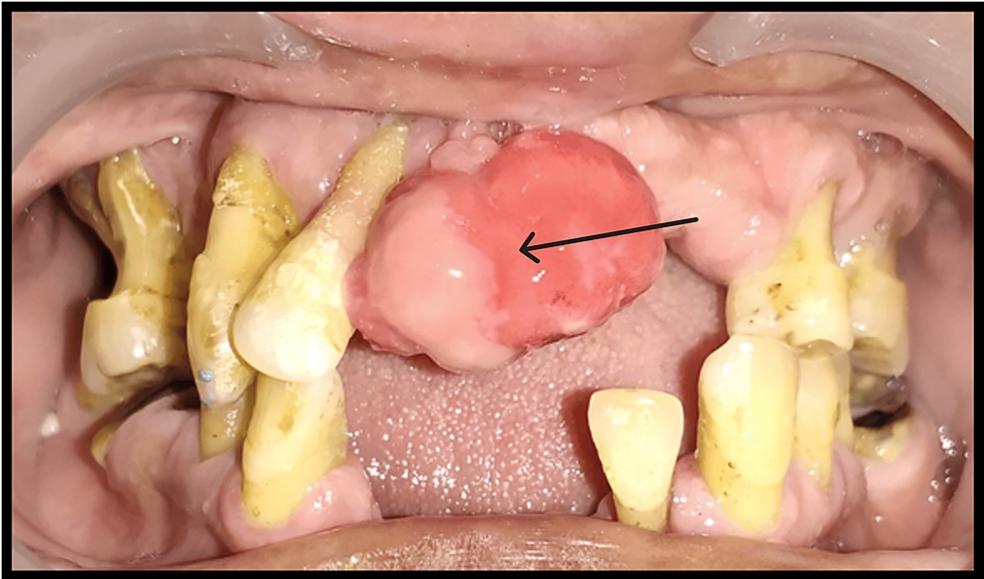
Intra-oral photograph (preoperative clinical photograph)

On intra-oral examination (Figure 1), a pedunculated growth was seen in the 11 and 12 regions. On palpation, swelling was hard in consistency, and no pain on palpation, no associated bleeding, or suppuration was present. The swelling was firm, slow-growing, and sessile, which was covered by normal mucosa. Other intra-oral examinations included missing the upper and lower anterior, generalized clinical attachment (CAL) loss of 9-10 mm, and generalized probing pocket depth (PPD) of 6-7 mm. 

**Figure 2:**
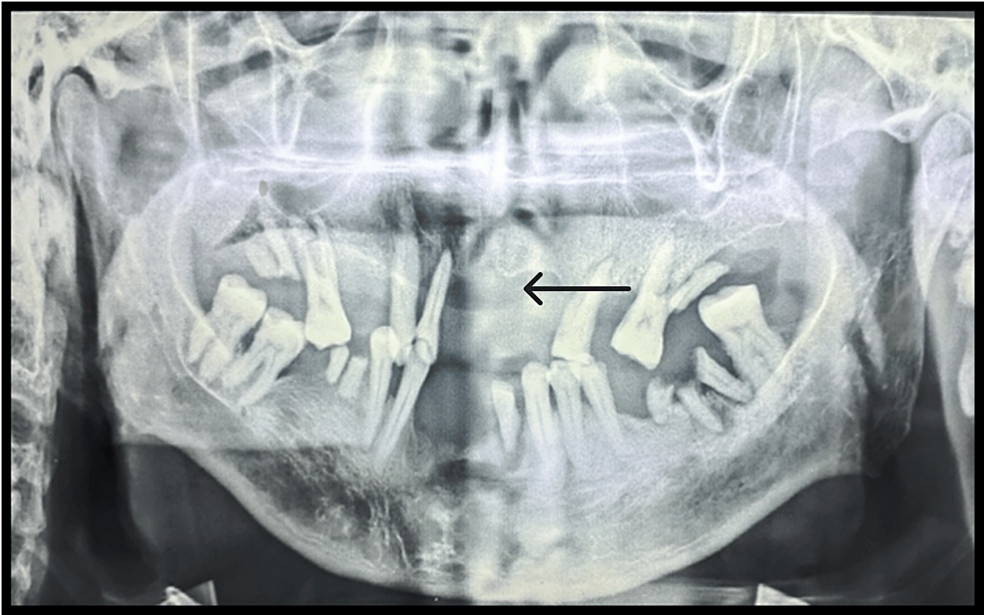
Preoperative orthopantomogram (OPG), bone loss seen in the maxillary anterior region.

The patient was then referred to the Department of Radiology for orthopantomogram (OPG) (Figure 2), which revealed generalized severe bone loss and bone destruction in the upper front region of the jaw. The patient was also referred to the Department of Oral Pathology for blood investigations, including hemoglobin (Hb), bleeding time (BT), and clotting time (CT). The results of the blood investigations are given in Table 1.

**Table 1:** Hematological findings Haemoglobin (Hb), bleeding time (BT), clotting time (CT), random blood sugar (RBS), minute (min), seconds (sec)

Hematology	Finding value	Normal value
Hb%	10.5 gm%	M: 12-15.5 gm%, F: 11-14.5 gm%
BT	1 min 30 sec	1-3 min
CT	2 min 21 sec	1-5 min
RBS	120 mg/dl	M: 79-160 mg/dl, F: 70-160 mg/dl

**Figure 3:**
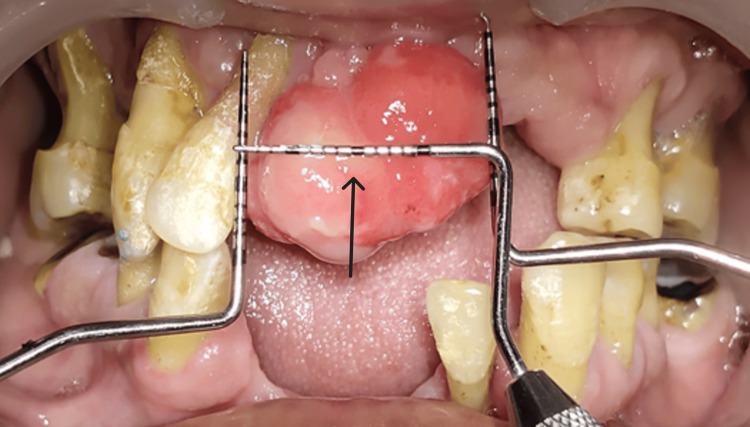
Preoperative photograph (preoperative clinical view showing the dimensions 20 x 15 mm)

**Figure 4:**
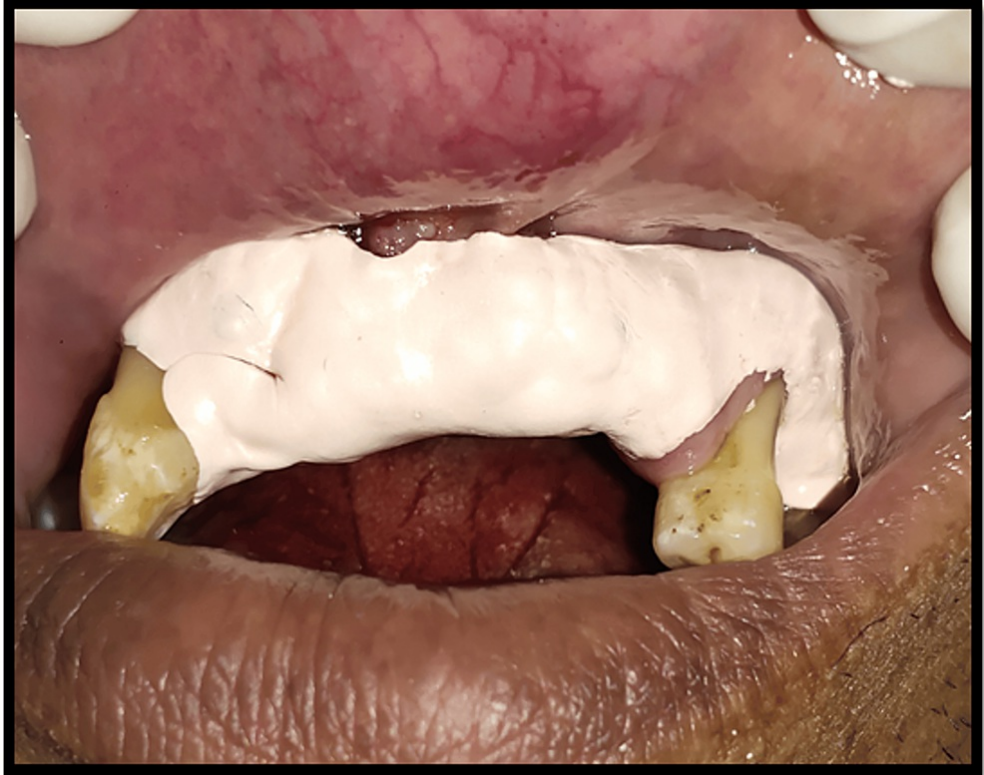
Periodontal pack placed after achieving hemostasis

Oral prophylaxis (ultrasonic scaling) was done. After seven days (Figure 3), the patient was recalled for surgical excision of the soft tissue growth. Under all aseptic conditions and precautions and under local anesthesia (lignocaine 2% and adrenaline), the excision of the overgrowth was done using a diode laser (Biolase EPIC- X Diode Laser, USA). Hemostasis was achieved using a pressure pack (gauze socked with local anesthesia) for 15 minutes. After achieving complete hemostasis, a dressing was given using a periodontal pack (Figure 4). The patient was recalled after seven days for follow-up, but the patient did not report for further appointments. 

**Figure 5:**
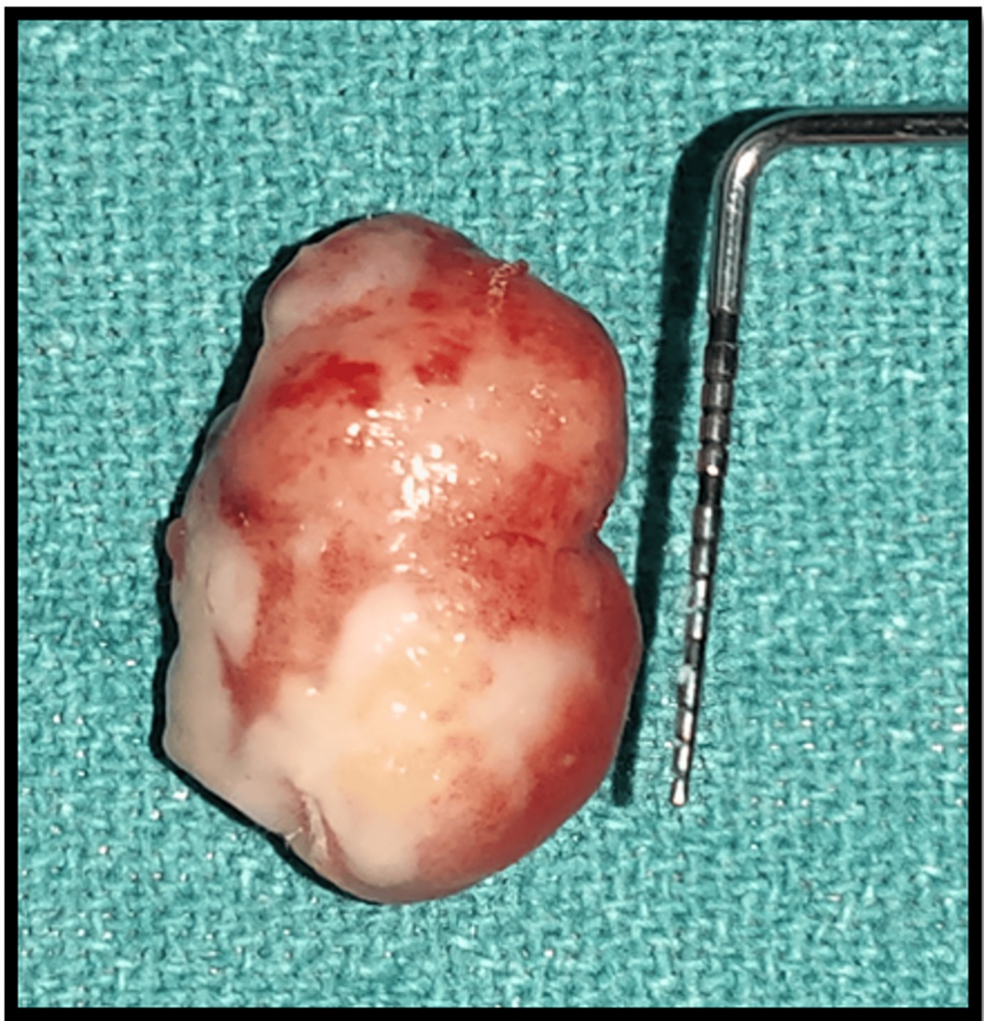
Excised tissue of 20 x 15 mm dimension

**Figure 6:**
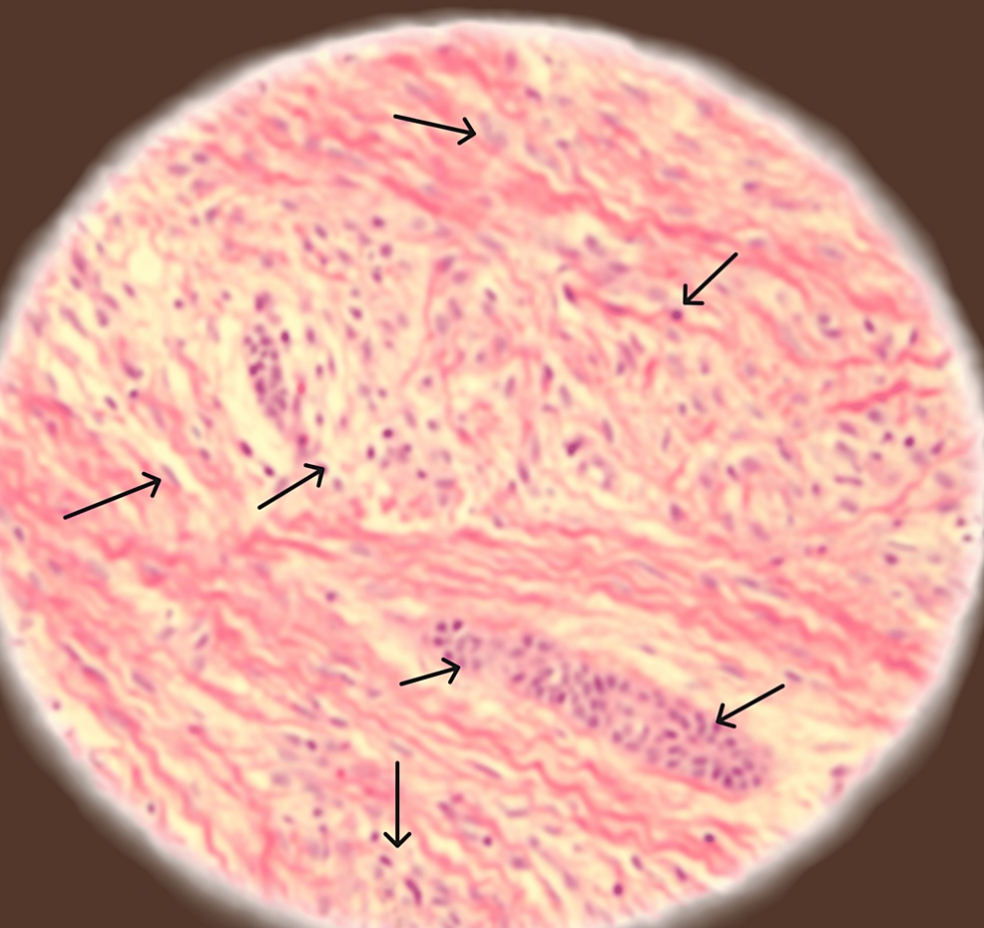
Histopathological photograph Arrows show interwoven fascicles of cellular fibrous connective tissues, and an island of odontogenic epithelium is scattered throughout the connective tissues; giant cells are seen in the periphery.

Postoperative instructions and medications, including amoxicillin 500 mg, aceclofenac (100.0 Mg) + serratiopeptidase (15.0 Mg) + paracetamol (325.0 Mg), for five days were given. The excised tissue (Figure 5) with measurement 20 x 15 mm, was preserved in a formalin-filled container and given to the Department of Oral Pathology for histopathological examination. The histopathological (Figure 6) examination revealed a proliferation of fibroblast with collagenous stroma and a comparatively cellular fibrous connective tissue with strands and remnants of dispersed inactive odontogenic epithelium. The histological diagnosis given was peripheral odontogenic fibroma.

## Discussion

Peripheral odontogenic fibromas usually have a satisfactory prognosis when treated primarily by localized excision. In peripheral odontogenic fibromas, calcified areas are rarely seen radiographically and usually do not impact the underlying bone, but in our case, the presence of periodontitis leads to evident bone loss. As part of the differential diagnosis, it is crucial to rule out additional inflammatory lesions, such as peripheral ossifying fibroma, giant cell fibroma, pyogenic granuloma, and peripheral giant cell granuloma when diagnosing peripheral odontogenic fibroma [4].

Michaelides, in his case report, described a case of peripheral odontogenic fibroma in the maxillary anterior region in which the recurrence was 34 months after excision. He concluded by pointing out the atypicality of the case and that only a few cases of recurrent peripheral odontogenic fibroma had been reported in the literature. Thus, in order to quickly identify potential recurrences, he recommended all patients with peripheral odontogenic fibroma to schedule routine follow-up visits [5]. Maheshwari et al., in their case report, concluded that even though peripheral odontogenic fibroma is a rare condition, it can clinically resemble a number of reactive, inflammatory, and malignant growths. Therefore, in order to prevent misdiagnosis related to this growth, a thorough history and meticulous histology are essential [6].

Armas et al., in their case report, revealed a three-time recurrence of peripheral odontogenic fibroma. The longest recorded time between recurrences was 11 years. The same was associated with a case of COF, despite the first two episodes not showing any radiographic findings. This highlights how crucial radiographic investigation and case follow-up are in such cases [7]. Patel et al., in their case report, recommended the resection of the periosteum for these tumors, because they found a greater recurrence rate in peripheral odontogenic fibroma when the periosteum was left intact during surgical excision of the tumor [8].

A case report by Pasalkar et al. presented a peripheral odontogenic fibroma in a 36-year-old female patient with an exophytic growth, which was lobulated pebbled-like on the right gingiva. Post-surgically, at six, 12, and 18 months, the follow-up of the patient showed no recurrence of the growth [9]. Eversole reported no recurrence [10]. Ritwik et al. found in their study that a reduced recurrence rate is linked to the presence of calcifications near odontogenic epithelial rests, while a higher recurrence rate is detected when surface epithelium with budding basal cell layers is present [11]. In this case report, recurrence was not recorded due to the poor follow-up by the patient.

## Conclusions

The normal presentation of a peripheral odontogenic fibroma is an exophytic mass that has an uncommon, benign, unencapsulated development. It has a smooth surface, a red or pink tint, and intermittent ulceration, and it can be sessile or pedunculated. Although it can appear anywhere in the jaw, it is usually found on the connected gingiva, especially in the molar and premolar regions. To avoid misdiagnosis, accurate investigations via radiographs and histological evaluation are essential. In addition, frequent patient monitoring is necessary to keep an eye out for any growth recurrence. This case report provides insights into the diagnosis, surgical intervention, and subsequent management of recurrence in this rare condition of peripheral odontogenic fibroma.
